# Supplementary material for: Hybrid Models and Biological Model Reduction with PyDSTool
Source: PLoS Comput Biol. 2012 Aug 9;8(8):e1002628. doi: 10.1371/journal.pcbi.1002628 (PMC3415397; doi:10.1371/journal.pcbi.1002628)
Supplement: Text S4 — Complete source code for the PyDSTool package (version 0.88.120504). Includes API documentation and help files linking to web pages. This file is identical to the current public release on Sourceforge.net. (ZIP) [file pcbi.1002628.s004.zip › PyDSTool/html/PyDSTool.ModelSpec'-pysrc.html]

xml version="1.0" encoding="ascii"?


PyDSTool.ModelSpec'


| Home | Trees | Indices | Help | PyDSTool |
| --- | --- | --- | --- | --- |

Package PyDSTool ::
Module ModelSpec'

[hide private]

[frames] | no frames]

# Source Code for Module PyDSTool.ModelSpec'

```
   1  """Structured model specification classes, and associated utilities.
 
   2  
 
   3  Robert Clewley, October 2005.
 
   4  
 
   5  
 
   6   Technical notes for ModelSpec attributes:
 
   7   _componentNameMap: object -> full (hierarchical) objname
 
   8   _registry: objname -> regObject:
 
   9                        obj, objLocalName, objClassName, objParentName
 
  10   _componentTypeMap: classname -> [objlist]
 
  11  """ 
  12  
 
  13  # ----------------------------------------------------------------------------
 
  14  
 
  15  from __future__ import division 
  16  from common import * 
  17  from utils import info as utils_info 
  18  from parseUtils import * 
  19  from errors import * 
  20  from Symbolic import * 
  21  
 
  22  from math import * 
  23  from utils import * 
  24  from numpy import Inf, NaN, isfinite,  mod, sum 
  25  from numpy import sometrue, alltrue 
  26  from copy import copy, deepcopy 
  27  import math, random 
  28  
 
  29  
 
  30  # ---------------------------------------------------------------------------
 
  31  ### Exports
 
  32  
 
  33  _classes = ['ModelSpec', 'Component', 'LeafComponent',
 
  34              'nameResolverClass'] 
  35  
 
  36  _functions = ['searchModelSpec', 'matchSubName', 'processMacro',
 
  37                'resolveMacroTargets'] 
  38  
 
  39  _objects = ['nameResolver'] 
  40  
 
  41  
 
  42  __all__ = _classes + _functions + _objects 
  43  
 
  44  # -----------------------------------------------------------------------------
 
  45  
 


46 -class ModelSpec(object):


47      """Model specification abstract class.
 
  48      Sub-classes can override targetLangs, compatibleSubcomponents,
 
  49      compatibleContainers, and compatibleGens.""" 
  50  
 
  51      targetLangs = () 
  52      compatibleSubcomponents = () 
  53      compatibleContainers = () 
  54      compatibleGens = () 
  55  
 


56 -    def __init__(self, name):


57          """Called by all sub-classes - do not override""" 
  58          self._registry = {} 
  59          self._componentNameMap = {} 
  60          self._componentTypeMap = {} 
  61          self.multiDefRefs = {} 
  62          self.funcSpecDict = {} 
  63          self.flatSpec = {'FScompatibleNames': symbolMapClass(),
 
  64                           'FScompatibleNamesInv': symbolMapClass()} 
  65          self.variables = {} 
  66          self.pars = {} 
  67          self.inputs = {} 
  68          self.auxfns = {} 
  69          self.components = {} 
  70          self.name = name 
  71          self.eventPars = [] 
  72          # Find unique list of all compatible subcomponent types inherited
 
  73          # from ModelSpec and its sub-classes. Include only the most specialized
 
  74          # sub-class of each ancestry.
 
  75          self._allSubcomponentTypes = allCompTypes(accrueCompTypes(self.__class__)) 
  76          # optional store for names of components that depend on this component's
 
  77          # variables -- needed for supporting network 'graph' abstractions
 
  78          # (in particular, to support edges)
 
  79          self.connxnTargets = [] 
  80          # list of unbound symbols
 
  81          self.freeSymbols = [] 
  82          # for use by ModelLibrary classes to identify interchangeable types of spec
 
  83          self.library_tag = '' 
  84          # in case a previous instance of this model spec existed in this
 
  85          # session, we need to clear out its associated names in
 
  86          # the nameResolverClass instance.
 
  87          nameResolver.clear(self) 
  88          self.validate()

  89  
 
  90  
 


91 -    def rename(self, newName):


92          oldName = self.name 
  93          for robj in self._registry.values(): 
  94              if robj.objParentName == oldName: 
  95                  robj.objParentName = newName 
  96          self.name = newName

  97  
 
  98  
 


99 -    def compileFuncSpec(self):


100          raise NotImplementedError("Only call this method on a concrete "
 
 101                                    "sub-class")

 102  
 
 103  
 


104 -    def flattenSpec(self, multiDefUnravel=True, globalRefs=None,
 
 105                      ignoreInputs=False, force=False):


106          """Flatten structured model specification to dictionary compatible with
 
 107         FuncSpec instantiation.
 
 108  
 
 109         Use globalRefs option to declare global variables used in the
 
 110         definitions that are not defined in them (e.g. time).
 
 111  
 
 112         Default for multiple quantity definitions is to unravel them.
 
 113         Use multiDefUnravel=False to override this.
 
 114  
 
 115         Use force option to rebuild flat spec if an existing one is
 
 116         out of date.
 
 117         """ 
 118  ##        Use the optional multiDefUnravel dictionary to selectively retain
 
 119  ##        compact specification or only partially unravel the definitions
 
 120  ##        during the flattening process. Use {} to force all definitions to
 
 121  ##        remain compact, otherwise the syntax is:
 
 122  ##
 
 123  ##             key = a rootname of multiple quantity names
 
 124  ##         --> value = either (a): pair (i1,i2) to unravel all indices of the
 
 125  ##                       rootname from i1 to i2 inclusive, or
 
 126  ##                     (b): a list of such pairs (the union of the intervals will
 
 127  ##                                                be used if they overlap), or
 
 128  ##                     (c): an empty list, so that the definition will remain
 
 129  ##                       compact."""
 
 130          #self.validate()
 
 131          if not self.isDefined(True, ignoreInputs): 
 132              raise AssertionError("Model spec not fully defined") 
 133          if not self.isComplete(globalRefs): 
 134              if globalRefs is None: 
 135                  print "Unresolved symbols:",  self.freeSymbols 
 136              else: 
 137                  print "Unresolved symbols:", remain(self.freeSymbols, globalRefs) 
 138              raise ValueError("Cannot flatten incomplete functional "
 
 139                               "specification") 
 140          if self.funcSpecDict == {} or force: 
 141              try: 
 142                  self.compileFuncSpec(ignoreInputs=ignoreInputs) 
 143              except KeyboardInterrupt: 
 144                  raise 
 145              except: 
 146                  print "compileFuncSpec() failed" 
 147                  raise 
 148          fs = self.funcSpecDict 
 149          # name mappings to be compatible with FuncSpec.py and python variable
 
 150          # naming rules: i.e. "a_name.b.x3" (ModelSpec) <-> "a_name_b_x3" (FuncSpec/python)
 
 151          FScompatibleNames = {} 
 152          FScompatibleNamesInv = {} 
 153          for name in self._registry.keys(): 
 154              FScompatibleNames[name] = replaceSep(name) 
 155              FScompatibleNamesInv[replaceSep(name)] = name 
 156          # store for later use
 
 157          self._FScompatibleNames = FScompatibleNames 
 158          self._FScompatibleNamesInv = FScompatibleNamesInv 
 159          # process multi-quantity definitions, if used
 
 160          # TEMP -- multiDefUnravel can only be True or False
 
 161          if self.multiDefRefs != {}: 
 162              unravelDict = {}.fromkeys(self.multiDefRefs) 
 163              if multiDefUnravel: 
 164                  for k in unravelDict: 
 165                      # default is to entirely unravel all definitions
 
 166                      infotuple = self.multiDefRefs[k] 
 167                      unravelDict[k] = [(infotuple[1], infotuple[2])] 
 168  ##            if multiDefUnravel is None:
 
 169  ##                for k in unravelDict:
 
 170  ##                    # default is to entirely unravel all definitions
 
 171  ##                    infotuple = self.multiDefRefs[k]
 
 172  ##                    unravelDict[k] = [(infotuple[1], infotuple[2])]
 
 173  ##            else:
 
 174  ##                for k in unravelDict:
 
 175  ##                    unravelDict[k] = []
 
 176  ##                # if multiDefUnravel == {} then the following will not change
 
 177  ##                # unravelDict from being all []
 
 178  ##                for k, v in multiDefUnravel.iteritems():
 
 179  ##                    if isinstance(v, list):
 
 180  ##                        unravelDict[k] = []
 
 181  ##                        if v != []:
 
 182  ##                            # list of pairs
 
 183  ##                            for p in v:
 
 184  ##                                assert p[0] < p[1], "Pair must be ordered"
 
 185  ##                                unravelDict[k].append(p)
 
 186  ##                    else:
 
 187  ##                        # single pair
 
 188  ##                        assert len(p) == 2
 
 189  ##                        assert p[0] < p[1], "Pair must be ordered"
 
 190  ##                        unravelDict[k] = [p]
 
 191          # Any unravelDict entries with [], and those remaining after a
 
 192          # partial unravelling, should still have their funcSpecDict
 
 193          # entry show a valid multiDef name, e.g. z[i,1,5] including i1
 
 194          # and i2
 
 195          outfs = {}  # output dictionary for funcspec initialization 
 196          quants = ['vars', 'pars', 'inputs'] 
 197          outfs['domains'] = {} 
 198          outfs['spectypes'] = {} 
 199          # mathNameMap maps title case math function names to all lower-case
 
 200          mathNameMap = dict(zip(allmathnames_symbolic,allmathnames)) 
 201          # ----- start of add_to_fs function -----
 
 202          def add_to_fs(k, name, carg, i=None): 
 203              # locally-defined because refers to outfs from scope of method
 
 204              # 'flattenSpec'
 
 205              c = copy(carg) 
 206              if 'for' in c.usedSymbols: 
 207                  # Activate macro for variable definition.
 
 208                  # Correct syntax already checked in Quantity __init__
 
 209                  # 'for' may have been used more than once
 
 210                  c, dummy1, dummy2 = processMacro(c, self) #._registry) 
 211                  # dummy1 and 2 are unused here
 
 212              c.mapNames(mathNameMap) 
 213              fsname = FScompatibleNames[name] 
 214              if i is None: 
 215                  try: 
 216                      outfs[k][fsname] = replaceSep(c.spec) 
 217                  except KeyError: 
 218                      outfs[k] = {fsname: replaceSep(c.spec)} 
 219              else: 
 220                  # c is a multi-quantity def and needs evaluating at i
 
 221                  try: 
 222                      outfs[k][fsname] = replaceSep(c[i]) 
 223                  except KeyError: 
 224                      outfs[k] = {fsname: replaceSep(c[i])} 
 225              outfs['domains'][fsname] = c.domain 
 226              if k not in ['pars', 'inputs']: 
 227                  # not necessary for pars and inputs
 
 228                  # as they must be explicitly defined
 
 229                  outfs['spectypes'][fsname] = c.spec.specType

 230          # ----- end of add_to_fs function -----
 
 231          full_mref_names = {} 
 232          if multiDefUnravel: 
 233              # reconstruct list of full names for mrefs, e.g. 'z[i]' from 'z'
 
 234              # otherwise just leave the dict empty
 
 235              for k, v in self.multiDefRefs.iteritems(): 
 236                  try: 
 237                      ksubs = FScompatibleNames[k] 
 238                  except KeyError: 
 239                      ksubs = replaceSep(k) 
 240                      FScompatibleNames[k] = ksubs 
 241                      FScompatibleNamesInv[ksubs] = k 
 242                      # also add all instances of the multiref to
 
 243                      # FScompatibleNames, so add z0 ... z10 for
 
 244                      # z[i,0,10].
 
 245                      for ix in range(v[1], v[2]+1): 
 246                          FScompatibleNames[k+str(ix)] = ksubs+str(ix) 
 247                          FScompatibleNamesInv[ksubs+str(ix)] = k+str(ix) 
 248                  full_mref_names[ksubs+"["+v[0]+","+str(v[1])+","+str(v[2])+"]"]\
 
 249                                 = ksubs 
 250          for k, v in fs.iteritems(): 
 251              if k in quants: 
 252                  for c in v: 
 253                      if c.name in full_mref_names.keys(): 
 254                          # unravel defs given by pairs p in unravelDict
 
 255                          root = full_mref_names[c.name] 
 256                          for p in unravelDict[root]: 
 257                              for i in xrange(p[0],p[1]+1): 
 258                                  name = root + str(i) 
 259                                  try: 
 260                                      if name not in outfs[k]: 
 261                                          add_to_fs(k, name, c, i) 
 262                                  except KeyError: 
 263                                      add_to_fs(k, name, c, i) 
 264                      else: 
 265                          add_to_fs(k, c.name, c) 
 266              elif k == 'auxfns': 
 267                  for a in v: 
 268                      afsname = FScompatibleNames[a.name] 
 269                      acopy = copy(a) 
 270                      acopy.mapNames(mathNameMap) 
 271                      aspec = acopy.spec 
 272                      try: 
 273                          outfs[k][afsname] = (a.signature, replaceSep(aspec)) 
 274                      except KeyError: 
 275                          outfs[k] = {afsname: (a.signature, replaceSep(aspec))} 
 276              elif k == 'complete': 
 277                  # ignore this -- it's not needed in the flattened specification
 
 278                  pass 
 279              else: 
 280                  outfs[k] = v 
 281          outfs['FScompatibleNames'] = symbolMapClass(FScompatibleNames) 
 282          outfs['FScompatibleNamesInv'] = symbolMapClass(FScompatibleNamesInv) 
 283          self.flatSpec = outfs   # for future reference 
 284          return outfs

 285  
 


286 -    def isDefined(self, verbose=False, ignoreInputs=False):


287          defined = True  # initial value 
 288          if len(self.compatibleGens) == 0: 
 289              if verbose: 
 290                  print "'%s' ill defined: empty compatibleGens"%self.name 
 291              return False 
 292          if len(self.targetLangs) == 0: 
 293              if verbose: 
 294                  print "'%s' ill defined: empty targetLangs"%self.name 
 295              return False 
 296          if self.isEmpty(): 
 297              if verbose: 
 298                  print "'%s' ill defined: empty contents"%self.name 
 299              return False 
 300          for v in self.variables.values(): 
 301              if not v.isDefined(verbose): 
 302                  if verbose: 
 303                      print "... in '%s' (type %s)"%(self.name,str(self.__class__)) 
 304                  return False 
 305          for p in self.pars.values(): 
 306              if not p.isDefined(verbose): 
 307                  if verbose: 
 308                      print "... in '%s' (type %s)"%(self.name,str(self.__class__)) 
 309                  return False 
 310          if not ignoreInputs: 
 311              for i in self.inputs.values(): 
 312                  if not i.isDefined(verbose): 
 313                      if verbose: 
 314                          print "... in '%s' (type %s)"%(self.name,str(self.__class__)) 
 315                          return False 
 316          for a in self.auxfns.values(): 
 317              if not a.isDefined(verbose): 
 318                  if verbose: 
 319                      print "... in '%s' (type %s)"%(self.name,str(self.__class__)) 
 320                  return False 
 321          return defined

 322  
 
 323  
 


324 -    def addConnxnTarget(self, targ):


325          if isinstance(targ, str): 
 326              if targ not in self.connxnTargets: 
 327                  self.connxnTargets.append(targ) 
 328          elif isinstance(targ, list): 
 329              for t in targ: 
 330                  if t not in self.connxnTargets: 
 331                      self.connxnTargets.append(t) 
 332          else: 
 333              raise TypeError("Invalid ModelSpec type for targ argument")

 334  
 
 335  
 


336 -    def delConnxnTarget(self, targ):


337          if isinstance(targ, str): 
 338              try: 
 339                  self.connxnTargets.remove(targ) 
 340              except ValueError: 
 341                  raise ValueError("Connection target %s not found"%targ) 
 342          elif isinstance(targ, list): 
 343              for t in targ: 
 344                  try: 
 345                      self.connxnTargets.remove(t) 
 346                  except ValueError: 
 347                      raise ValueError("Connection target %s not found"%t)

 348  
 
 349  
 


350 -    def __contains__(self, obj):


351          try: 
 352              if obj.name in self._registry: 
 353                  return obj == self._registry[obj.name].obj 
 354              elif self.multiDefInfo[0]: 
 355                  # there are multiply-defined variables here
 
 356                  # if subject is a single Quantity that is defined here
 
 357                  # in multiple definition form.
 
 358                  return isMultiDefClash(obj, self.multiDefRefs) 
 359              else: 
 360                  return False 
 361          except AttributeError: 
 362              return False

 363  
 
 364  
 


365 -    def __eq__(self, other, diff=False):


366          results = [] 
 367          try: 
 368              results.append(type(self) == type(other)) 
 369              results.append(self.name == other.name) 
 370              results.append(self._registry == other._registry) 
 371          except AttributeError, e: 
 372              if diff: 
 373                  print "Type:", className(self), results 
 374                  print "  " + e 
 375              return False 
 376          if diff: 
 377              print "Type:", className(self), results 
 378          return alltrue(results)

 379  
 
 380  
 


381 -    def __ne__(self, other):


382          return not self.__eq__(other)

 383  
 
 384  
 


385 -    def difference(self, other):


386          """Print the difference between two ModelSpecs to screen.""" 
 387          self.__eq__(other, diff=True)

 388  
 
 389  
 


390 -    def validate(self):


391          assert isinstance(self.name, str), "ModelSpec name must be a string" 
 392          assert len(remain(self.targetLangs, targetLangs)) == 0, \
 
 393                 "Invalid target language for '" + self.name + "'" 
 394          if hasattr(self, 'components'): 
 395              # leaf nodes do not have this
 
 396              for c in self.components.values(): 
 397                  assert c in self._componentNameMap, "Component name map incomplete" 
 398  #                assert remain(self._componentNameMap[c],
 
 399  #                              self._registry.keys()) == []
 
 400          for v in self.flatSpec['FScompatibleNamesInv'](self.variables.values()): 
 401              assert v in self._componentNameMap, "Variable name map incomplete" 
 402          for p in self.flatSpec['FScompatibleNamesInv'](self.pars.values()): 
 403              if p not in self._componentNameMap and p.name not in self.eventPars: 
 404                  print "\n", self.name, self 
 405                  print "Pars:", p 
 406                  print p.name in self.flatSpec['FScompatibleNames'] 
 407                  print p.name in self.flatSpec['FScompatibleNamesInv'] 
 408                  print self.eventPars 
 409                  print self.flatSpec['FScompatibleNamesInv'](p) 
 410                  print "Component name map:", self._componentNameMap 
 411                  raise AssertionError("Parameter name map incomplete") 
 412          i = None 
 413          for i in self.flatSpec['FScompatibleNamesInv'](self.inputs.values()): 
 414              assert i in self._componentNameMap, "External input name map incomplete" 
 415          for a in self.flatSpec['FScompatibleNamesInv'](self.auxfns.values()): 
 416              assert a in self._componentNameMap, "Auxiliary function name map incomplete"

 417          # !! check that compatibleGens point to known Generator types !!
 
 418          # if get this far without raising exception, then valid
 
 419  
 
 420  
 


421 -    def add(self, arg, tosubcomponent=None):


422          """Add object to registry, into a specified sub component if
 
 423          provided.
 
 424          """ 
 425          if isinstance(arg, list): 
 426              for obj in arg: 
 427                  self.add(obj, tosubcomponent) 
 428          elif tosubcomponent is not None: 
 429              if tosubcomponent in self.components: 
 430                  c = deepcopy(self[tosubcomponent]) 
 431                  self.remove(tosubcomponent) 
 432                  c.add(arg) 
 433                  self.add(c) 
 434              elif tosubcomponent == '': 
 435                  # no subcomponent, add to top level
 
 436                  self.add(arg) 
 437              else: 
 438                  raise ValueError("Unknown sub-component %s"%tosubcomponent) 
 439          elif isHierarchicalName(arg.name): 
 440              # add the object to the appropriate sub-component
 
 441              obj = deepcopy(arg) 
 442              hier_name_split = obj.name.split(NAMESEP) 
 443              comp_name = hier_name_split[0] 
 444              rest_name = ".".join(hier_name_split[1:]) 
 445              obj.rename(rest_name) 
 446              self.add(obj, tosubcomponent=comp_name) 
 447          else: 
 448              obj = deepcopy(arg) 
 449              objname = self._register(obj) 
 450              if isinstance(obj, Var): 
 451                  self.variables[objname] = obj 
 452              elif isinstance(obj, Par): 
 453                  self.pars[objname] = obj 
 454              elif isinstance(obj, Input): 
 455                  self.inputs[objname] = obj 
 456              elif isinstance(obj, Fun): 
 457                  self.auxfns[objname] = obj 
 458              elif isinstance(obj, ModelSpec): 
 459                  if not issubclass(self.__class__, obj.compatibleContainers): 
 460                      print "Component " + self.name + ": " + self.__class__.__name__ 
 461                      print "Invalid sub-component " + objname \
 
 462                            + " (type " + className(obj) + ") to add," 
 463                      print "  with compatible container types:", obj.compatibleContainers 
 464                      print "Compatible sub-component types: ", self._allSubcomponentTypes 
 465                      raise ValueError("Incompatible sub-component type"
 
 466                                       " for object '" + objname + "'") 
 467                  else: 
 468                      self.components[objname] = obj 
 469              else: 
 470                  raise TypeError("Invalid Quantity object to add")

 471  
 
 472  
 


473 -    def isEmpty(self):


474          raise NotImplementedError("Only call this method on a concrete "
 
 475                                    "sub-class")

 476  
 
 477  
 


478 -    def _register(self, obj, depth=0, parent_obj=None):


479          # depth = recursion depth, when this component registers its
 
 480          # subcomponents' definitions (from their _registry attributes)
 
 481          if len(obj.compatibleGens) == 0: 
 482              # object inherits self's
 
 483              obj.compatibleGens = self.compatibleGens 
 484          elif len(remain(obj.compatibleGens, self.compatibleGens)) > 0: 
 485              print remain(obj.compatibleGens, self.compatibleGens) 
 486              raise ValueError("Incompatible generators found in component"
 
 487                               " '" + obj.name + "'") 
 488          if len(obj.targetLangs) == 0: 
 489              # object inherits self's
 
 490              obj.targetLangs = self.targetLangs 
 491          elif len(remain(obj.targetLangs, self.targetLangs)) > 0: 
 492              print remain(obj.targetLangs, self.targetLangs) 
 493              raise ValueError("Incompatible target language in component"
 
 494                               " '" + obj.name + "'") 
 495          if obj.name in protected_allnames: 
 496              raise ValueError("Name '" + obj.name + "' is a protected name") 
 497          if not compareClassAndBases(obj, self._allSubcomponentTypes): 
 498              print "Valid sub-component types that have been declared:", \
 
 499                    self._allSubcomponentTypes 
 500              raise TypeError("Invalid type for object '" + obj.name + \
 
 501                              "' in component '" + self.name + "'") 
 502          if parent_obj is None: 
 503              parentname = None 
 504          else: 
 505              parentname = parent_obj.name 
 506          if isMultiDefClash(obj, self.multiDefRefs, parentname): 
 507              raise ValueError("Object %s defines names that already exist in"
 
 508                               " registry"%obj.name) 
 509  #        # TEMP
 
 510  #        print "\n------------------------"
 
 511  #        print depth, self.name, self.freeSymbols
 
 512          if hasattr(obj, '_registry'): 
 513              # if obj is a Component, then process its registry into ours
 
 514              # but register the object itself
 
 515              do_obj_register = False 
 516              # globalized name means we must create a local namemap
 
 517              # for obj and its local free symbols.
 
 518              # namemap for a registry object maps original (local) object name
 
 519              # to globalized name at this level.
 
 520              # tracebacknames are the names of all sub-component registry
 
 521              # names that are to be added to the registry at this level.
 
 522              namemap = {} 
 523              tracebacknames = [] 
 524              for sub_regObj in obj._registry.values(): 
 525                  subobj = sub_regObj.obj 
 526                  subobjname = self._register(subobj, depth+1, obj) 
 527                  # _register won't have created an entry yet
 
 528                  namemap[subobj.name] = subobjname 
 529                  if isMultiRef(subobjname): 
 530                      # assumes isMultiRef(objname) == sub_regObj.obj.multiDefInfo[0]
 
 531                      # Add non-definition version of name to dictionary,
 
 532                      # i.e. add z to mapping dictionary containing z[i,0,4]
 
 533                      rootname = subobj.multiDefInfo[1] 
 534                      ixname = subobj.multiDefInfo[2] 
 535                      namemap[rootname] = obj.name+NAMESEP+rootname 
 536                  tracebacknames.append(subobjname) 
 537              # for all newly registered names, add their associated
 
 538              # namemap to the regObject, and map all occurrences of the
 
 539              # local names from the sub-component to their new, globalized
 
 540              # names at this level, now that we have collected up the namemap.
 
 541              #
 
 542              # First, take out symbols defined by obj (i.e. those appearing in
 
 543              # its registry) from self.freeSymbols
 
 544              mapper = symbolMapClass(namemap) 
 545              globalized_newdefs = mapper(obj._registry.keys()) 
 546              self.freeSymbols = remain(self.freeSymbols,
 
 547                                        globalized_newdefs) 
 548              obj_freeSymbols = mapper(obj.freeSymbols) 
 549  #            # TEMP
 
 550  #            print "New self.freeSymbols:", self.freeSymbols
 
 551  #            print "Processing ", obj.name
 
 552  #            print "Namemap"
 
 553  #            info(namemap)
 
 554  #            print "Obj free (orig):", obj.freeSymbols, "(mapped)", obj_freeSymbols
 
 555              # the following never executes!
 
 556              if obj_freeSymbols != obj.freeSymbols: 
 557                  print "mapped object free symbols not same as originals" 
 558                  # Test code introspection for HH_spectest.py
 
 559  #            if self.name == 'cell1' and obj.name == 'chan_s21':
 
 560  #                # this is None, so comment out namemap[s] = snew below!
 
 561  #                print "cell1: parentname =", parentname
 
 562              for s in obj.freeSymbols: 
 563                  if s not in namemap: 
 564                      # then it didn't get mapped, so add to namemap
 
 565                      # that will be embedded in reg object
 
 566                      if parentname is None: 
 567                          snew = self.name+NAMESEP+s 
 568                      else: 
 569                          snew = parentname+NAMESEP+s 
 570  #                    namemap[s] = snew
 
 571  #                    # TEMP
 
 572  #                    print self.name, obj.name, "namemap[%s] = %s"%(s,snew)
 
 573              for sub_regObj in obj._registry.values(): 
 574                  subobjname = namemap[sub_regObj.obj.name] 
 575                  subsplit = subobjname.split(NAMESEP) 
 576                  # resolve the first parent of objects brought up from deep
 
 577                  # in the sub-component tree
 
 578                  if len(subsplit) > 1: 
 579                      actual_parentname = subsplit[-2] 
 580                      if actual_parentname in obj.components: 
 581                          actual_parent = obj.components[actual_parentname] 
 582  #                        print "Old parent %s"%actual_parentname
 
 583  #                        print "parent is obj '%s': self=%s, subobj=%s"%(obj.name, self.name, subobjname)
 
 584  #                        if obj.name == 'cell1':
 
 585  #                            1/0
 
 586                      else: 
 587                          # TEMP
 
 588  #                        print "Changing parent from %s to %s"%(actual_parentname, self.name)
 
 589  #                        print "for obj %s, sub-obj %s"%(obj.name, subobjname)
 
 590  #                        print obj._registry.keys()
 
 591  #                        print obj.components.keys()
 
 592  #                        1/0
 
 593                          actual_parent = obj 
 594  #                        actual_parentname = self.name
 
 595  #                        actual_parent = self
 
 596  #                        print "parent is self '%s' (1): obj=%s, subobj=%s"%(self.name, obj.name, subobjname)
 
 597                  else: 
 598                      actual_parent = obj 
 599                  new_regObj = deepcopy(sub_regObj) 
 600                  if isinstance(new_regObj.obj, Quantity): 
 601                      if new_regObj.obj.typestr == 'auxfn': 
 602                          # do not map the signature variables
 
 603                          func_namemap = copy(namemap) 
 604                          for s in new_regObj.obj.signature: 
 605                              try: 
 606                                  del func_namemap[s] 
 607                              except KeyError: 
 608                                  pass 
 609                          new_regObj.obj.mapNames(func_namemap) 
 610                      else: 
 611                          new_regObj.obj.mapNames(namemap) 
 612                  self._registry[subobjname] = regObject(new_regObj.obj,
 
 613                                                         actual_parent) 
 614                  self._registry[subobjname].namemap = namemap 
 615                  # fix new registry object's local name to not include the
 
 616                  # highest level name prefix
 
 617                  localName = self._registry[subobjname].objLocalName 
 618                  temp = localName.split(NAMESEP) 
 619                  if len(temp) > 1: 
 620                      localName = NAMESEP.join(temp[1:]) 
 621                  self._registry[subobjname].objLocalName = localName 
 622              objname = obj.name 
 623              self._componentNameMap[obj] = objname #tracebacknames 
 624              oclasses = getSuperClasses(obj, ModelSpec) 
 625              for oclass in oclasses: 
 626                  try: 
 627                      if obj not in self._componentTypeMap[oclass]: 
 628                          self._componentTypeMap[oclass].append(obj) 
 629                  except KeyError: 
 630                      self._componentTypeMap[oclass] = [obj] 
 631              do_free_symbs = True 
 632          else: 
 633              # obj is a Quantity object
 
 634              do_obj_register = (depth == 0) 
 635              do_free_symbs = do_obj_register 
 636              try: 
 637                  if depth > 0: # and obj.scope == localscope: 
 638                      objname = nameResolver(obj, self, parentname) 
 639                  else: 
 640                      objname = obj.name 
 641              except AttributeError: 
 642                  raise TypeError("Invalid object type in _register(): %s (type"
 
 643                                  " %s)"%(obj.name, className(obj))) 
 644  #            # TEMP
 
 645  #            print "Processing Quant:", obj.name, "(%s)"%objname
 
 646              if hasattr(obj, 'multiDefInfo'): 
 647                  # then Quantity spec may be defining multiple quantities,
 
 648                  # so check it
 
 649                  if obj.multiDefInfo[0]: 
 650                      # no longer need first entry of multiDefInfo.
 
 651                      # store the info for purposes of flattening, using
 
 652                      # the *rootname* as key (e.g. 'z' and not 'z[i]').
 
 653                      # Note that the rootname has the parentname appended,
 
 654                      # if it exists
 
 655                      if parentname is None: 
 656                          rootname = obj.multiDefInfo[1] 
 657                      else: 
 658                          rootname = parentname+NAMESEP+obj.multiDefInfo[1] 
 659                      self.multiDefRefs[rootname] = obj.multiDefInfo[2:] 
 660              self._componentNameMap[obj] = objname 
 661              oclasses = getSuperClasses(obj) 
 662              for oclass in oclasses: 
 663                  try: 
 664                      if obj not in self._componentTypeMap[oclass]: 
 665                          self._componentTypeMap[oclass].append(obj) 
 666                  except KeyError: 
 667                      self._componentTypeMap[oclass] = [obj] 
 668              if objname in self._registry: 
 669                  raise ValueError("Name '%s' already exists in "%objname \
 
 670                                   + "registry of object '%s'"%self.name) 
 671              # remove free symbols from self's sub-components if
 
 672              # they are being defined here
 
 673              for fs in copy(self.freeSymbols): 
 674                  fs_split = fs.split(NAMESEP) 
 675                  if len(fs_split) > 1: 
 676                      checkname = "".join(fs_split[1:]) 
 677                  else: 
 678                      checkname = fs 
 679                  if objname == checkname: 
 680                      self.freeSymbols.remove(fs) 
 681              obj_freeSymbols = obj.freeSymbols 
 682          if do_obj_register: 
 683              self._registry[objname] = regObject(deepcopy(obj),
 
 684                                                  parent_obj or self) 
 685              # localname = objname so no need for the next line
 
 686  #            self._registry[objname].objLocalName = obj.name.split(NAMESEP)[-1]
 
 687          if do_free_symbs: 
 688  #            # TEMP
 
 689  #            print "new free symbols from obj:", obj_freeSymbs
 
 690  #            print "defined:", self._registry.keys()
 
 691              self.freeSymbols.extend(remain(obj_freeSymbols,
 
 692                                             self._registry.keys()+self.freeSymbols)) 
 693  #            print "resulting self.free:", self.freeSymbols
 
 694          # return objname in case _register is being called recursively
 
 695          return objname

 696  
 
 697  
 


698 -    def __delitem__(self, name):


699          """Delete named object (Var, Par, Fun, Input, or sub-component)
 
 700          """ 
 701          self.remove(name)

 702  
 


703 -    def __getitem__(self, name):


704          """Return object named using the hierarchical naming format.""" 
 705          try: 
 706              return self._registry[name].obj 
 707          except KeyError: 
 708              # full hierarchical name not found in registry, so
 
 709              # check for components (not kept in registry)
 
 710              name_list = name.split('.') 
 711          try: 
 712              c = self.components[name_list[0]] 
 713          except KeyError: 
 714              if name == '': 
 715                  # for compatibility with empty string index
 
 716                  return self 
 717              else: 
 718                  raise ValueError("Object %s not found in registry"%name) 
 719          else: 
 720              rest = name_list[1:] 
 721              if len(rest) > 0: 
 722                  return c['.'.join(rest)] 
 723              else: 
 724                  return c

 725  
 
 726  
 


727 -    def remove(self, target):


728          """Remove target component from specification.
 
 729  
 
 730         Use global hierarchical names for components if specifying a string.""" 
 731  
 
 732          if hasattr(target, 'name'): 
 733              objname = target.name 
 734          elif isinstance(target, str): 
 735              objname = target 
 736          elif isinstance(target, list): 
 737              for t in target: self.remove(t) 
 738              return 
 739          else: 
 740              raise TypeError("Invalid type to remove from ModelSpec "
 
 741                              "'%s'"%self.name) 
 742          try: 
 743              del self._registry[objname] 
 744          except KeyError: 
 745              # objname may refer to a component, which is not stored in registry
 
 746              pass 
 747          if isHierarchicalName(objname): 
 748              tempname = objname.split(NAMESEP) 
 749              parentname = tempname[0] 
 750              try: 
 751                  self.components[parentname].remove("".join(tempname[1:])) 
 752              except ValueError, e: 
 753                  print e 
 754                  raise ValueError("Error recognizing parent object '%s' in "
 
 755                                   "sub-component '%s'"%(parentname,objname)) 
 756          else: 
 757              if objname in self.variables: 
 758                  del self.variables[objname] 
 759              elif objname in self.pars: 
 760                  del self.pars[objname] 
 761              elif objname in self.inputs: 
 762                  del self.inputs[objname] 
 763              elif objname in self.components: 
 764                  del self.components[objname] 
 765              elif objname in self.auxfns: 
 766                  del self.auxfns[objname] 
 767                  protected_auxnamesDB.removeAuxFn(objname) 
 768              else: 
 769                  raise ValueError("%s: Error recognizing object '%s'"%(self.name,objname)) 
 770          # This is inefficient, but simply reregister everything to
 
 771          # work out the change in free symbols!
 
 772          self.freeSymbols = [] 
 773          self.multiDefRefs = {} 
 774          self._registry = {} 
 775          self._componentNameMap = {} 
 776          self._componentTypeMap = {} 
 777          nameResolver.clear(self) 
 778          for v in self.variables.values(): 
 779              self._register(v) 
 780          for p in self.pars.values(): 
 781              self._register(p) 
 782          for i in self.inputs.values(): 
 783              self._register(i) 
 784          for a in self.auxfns.values(): 
 785              self._register(a) 
 786          # do components, if any, last, so that self._componentNameMap is
 
 787          # built up properly (otherwise get validation error)
 
 788          if hasattr(self, 'components'): 
 789              # leaf nodes do not have this
 
 790              for c in self.components.values(): 
 791                  self._register(c) 
 792          try: 
 793              self.validate() 
 794          except AssertionError, e: 
 795              raise RuntimeError("Model spec structure inconsistent: "+str(e))

 796  
 
 797  
 


798 -    def isComplete(self, globalRefs=None):


799          if globalRefs is None: 
 800              globalRefs = allmathnames_symbolic + \
 
 801                    ['True', 'False', 'and', 'or', 'not'] 
 802          else: 
 803              globalRefs.extend(allmathnames_symbolic + \
 
 804                                ['True', 'False', 'and', 'or', 'not']) 
 805          return remain(self.freeSymbols, globalRefs) == []

 806  
 
 807  
 


808 -    def __call__(self):


809          # info is defined in PyDSTool.utils
 
 810          utils_info(self.__dict__, "ModelSpec " + self.name)

 811  
 
 812  
 


813 -    def info(self, verboselevel=1):


814          if verboselevel > 0: 
 815              # info is defined in PyDSTool.utils
 
 816              utils_info(self.__dict__, "ModelSpec " + self.name,
 
 817                         recurseDepthLimit=1+verboselevel) 
 818          else: 
 819              print self.__repr__()

 820  
 
 821  
 


822 -    def __repr__(self):


823          return self._infostr(1)

 824  
 


825 -    def _infostr(self, verbose=1):


826          base_str = "Component " + self.name 
 827          if verbose > 0: 
 828              if len(self.components) > 0: 
 829                  base_str += "\n Components: ( " 
 830                  base_str += ", ".join([c._infostr(verbose-1) for c in self.components.values()]) + " )" 
 831              if len(self.variables) > 0: 
 832                  base_str += "\n Variables: ( " 
 833                  base_str += ", ".join([str(c) for c in self.variables.values()]) + " )" 
 834              if len(self.pars) > 0: 
 835                  base_str += "\n Parameters: ( " 
 836                  base_str += ", ".join([str(c) for c in self.pars.values()]) + " )" 
 837              if len(self.inputs) > 0: 
 838                  base_str += "\n Inputs: ( " 
 839                  base_str += ", ".join([str(c) for c in self.inputs.values()]) + " )" 
 840              if len(self.auxfns) > 0: 
 841                  base_str += "\n Functions: ( " 
 842                  base_str += ", ".join([str(c) for c in self.auxfns.values()]) + " )" 
 843          return base_str

 844  
 
 845  
 


846 -    def search(self, name, component_type_order=None):


847          """Find Quantity objects containing a component named <name>,
 
 848          of type given by the hierarchical name <comptype1.comptype2. ... .name>,
 
 849          where component_type_order = [<comptype1>, <comptype2>, ... ],
 
 850          and <comptypeN> may be a component type (as a wildcard) or a specific
 
 851          component name.
 
 852          """ 
 853          return searchModelSpec(self, name, component_type_order)

 854  
 
 855  
 
 856      __str__ = __repr__ 
 857  
 
 858  
 


859 -    def __copy__(self):


860          pickledself = pickle.dumps(self) 
 861          return pickle.loads(pickledself)

 862  
 
 863  
 


864 -    def __deepcopy__(self, memo=None, _nil=[]):


865          pickledself = pickle.dumps(self) 
 866          return pickle.loads(pickledself)

 867  
 
 868  
 
 869  # ----------------------------------------------------------------------------
 
 870  
 


871 -class Component(ModelSpec):


872      """Non-leaf node sub-class of ModelSpec abstract class.""" 
 873  
 


874 -    def compileFuncSpec(self, ignoreInputs=False):


875          self.validate() 
 876          assert self.isDefined(True, ignoreInputs=ignoreInputs), \
 
 877                 "Node '" + self.name + "' is not completely defined" 
 878          fsdict = {} 
 879          fsdict['inputs'] = [] 
 880          fsdict['vars'] = [] 
 881          fsdict['pars'] = [] 
 882          fsdict['auxfns'] = [] 
 883          for objname, regObj in self._registry.iteritems(): 
 884              objtype = regObj.obj.typestr+'s' 
 885              add_obj = regObj.obj.renderForCode() 
 886              if regObj.namemap != {}: 
 887                  if isinstance(add_obj, Fun): 
 888                      # AuxFns may only reference pars, so raise
 
 889                      # error if the auxfn's namemap refers (i.e. is bound)
 
 890                      # to a non-parameter
 
 891                      for name in regObj.namemap: 
 892                          mappedName = regObj.namemap[name] 
 893                          if mappedName in add_obj.freeSymbols and not \
 
 894                             isinstance(self._registry[mappedName].obj, (Par, Fun)): 
 895                              print "Problem with registry object:", \
 
 896                                    self._registry[mappedName].obj 
 897                              print "in auxiliary function:", add_obj.name 
 898                              print "   that has free symbols:", \
 
 899                                    add_obj.freeSymbols 
 900                              raise TypeError("Fun '" + add_obj.name + "' "
 
 901                                              "cannot bind symbols to non-"
 
 902                                              "pars (info printed above)") 
 903                  # redundant mapping (already has been done)
 
 904  #                add_obj.spec.mapNames(regObj.namemap)
 
 905              fsdict[objtype].append(add_obj) 
 906          fsdict['complete'] = self.isComplete() 
 907          self.funcSpecDict = fsdict

 908  
 
 909  
 


910 -    def isEmpty(self):


911          return self.components == {}

 912  
 
 913  
 


914 -    def isDefined(self, verbose=False, ignoreInputs=False):


915          # Quantity is defined if it has a specified definition
 
 916          # (even with unbound references)
 
 917          defined = ModelSpec.isDefined(self, verbose, ignoreInputs) 
 918          for c in self.components.values(): 
 919              if not c.isDefined(verbose, ignoreInputs): 
 920                  return False 
 921          return defined

 922  
 
 923  # ----------------------------------------------------------------------------
 
 924  
 


925 -class LeafComponent(ModelSpec):


926      """Leaf node sub-class of ModelSpec abstract class.""" 
 927  
 


928 -    def _register(self, obj, depth=0, parent_obj=None):


929          if not compareBaseClass(obj, Quantity): 
 930              print "Bad argument %s (type %s) to register"%(str(obj), type(obj)) 
 931              raise TypeError("Not a valid Quantity type") 
 932          return ModelSpec._register(self, obj, 0, parent_obj)

 933  
 
 934  
 


935 -    def isEmpty(self):


936          return self.variables == {} and self.pars == {} \
 
 937                 and self.inputs == {}

 938  
 
 939  
 


940 -    def compileFuncSpec(self, ignoreInputs=False):


941          self.validate() 
 942          assert self.isDefined(ignoreInputs=ignoreInputs), \
 
 943                 "Node '" + self.name + "' is not completely defined" 
 944          fsdict = {} 
 945          fsdict['vars'] = [deepcopy(v) for v in self.variables.values()] 
 946          fsdict['pars'] = [deepcopy(p) for p in self.pars.values()] 
 947          fsdict['inputs'] = [deepcopy(i) for i in self.inputs.values()] 
 948          fsdict['auxfns'] = [deepcopy(a) for a in self.auxfns.values()] 
 949          self.funcSpecDict = fsdict

 950  
 
 951  
 


952 -    def add(self, arg):


953          if isinstance(arg, list): 
 954              for obj in arg: 
 955                  self.add(obj) 
 956          else: 
 957              if isinstance(arg, ModelSpec): 
 958                  raise TypeError("Cannot add sub-components to a leaf node") 
 959              else: 
 960                  ModelSpec.add(self, arg)

 961  
 
 962  # add the valid sub-component types now that all classes have been declared
 
 963  Component.compatibleSubcomponents=(Par, Var, Input, Fun,
 
 964      Component, LeafComponent) 
 965  Component.compatibleContainers=(Component,) 
 966  
 
 967  LeafComponent.compatibleSubcomponents=(Par, Var, Input, Fun) 
 968  LeafComponent.compatibleContainers=(Component,LeafComponent) 
 969  
 
 970  # ----------------------------------------------------------------------------
 
 971  ### Search utilities
 
 972  
 


973 -def allCompTypes(class_list):


974      """Filter full list of all super-classes' compatible sub-component
 
 975      types to remove any classes that are not over-ridden by sub-classes.
 
 976  
 
 977      e.g. If Par was allowed for Component type, but a sub-class of
 
 978      Component specifies only a sub-class of Par may be used, then
 
 979      Par is removed from the original list of valid component types.
 
 980      """ 
 981      filter_list = [] 
 982      for c in class_list: 
 983          filter_list.extend([d for d in class_list if issubclass(c, d) and c!=d]) 
 984      return [c for c in class_list if c not in filter_list]

 985  
 
 986  
 
 987  
 


988 -def accrueCompTypes(c):


989      """Collect all super-class component types (string names) and accumulate
 
 990      recursively.
 
 991      """ 
 992      cs = list(c.compatibleSubcomponents) 
 993      for cb in c.__bases__: 
 994          if cb is ModelSpec: 
 995              # don't go any deeper as there won't be any more
 
 996              # componentTypes -- this is the top of this class tree
 
 997              break 
 998          else: 
 999              try: 
1000                  cs.extend(accrueCompTypes(cb)) 
1001              except AttributeError: 
1002                  # cover the event of a user-defined multiple
 
1003                  # inheritance with non ModelSpec classes
 
1004                  pass 
1005      return makeSeqUnique(cs)

1006  
 
1007  
 


1008 -def matchSubName(nameList, subName, position, level=0, invert=False):


1009      """Return a list of names from nameList matching subName at the given
 
1010     position (at the hierarchical name level given, if the name is
 
1011     hierarchical).
 
1012  
 
1013     e.g. For a hierarchical variable name such as 'sRM.s_cell1_cell2',
 
1014     level=0 will cause a search for a match with subName in the top-level name,
 
1015     i.e. 'sRM', at the given position. Whereas, level=1 will search for a match
 
1016     in 's_cell1_cell2' at the given position.
 
1017  
 
1018     invert=True will return names that did not match.""" 
1019      matchList = [] 
1020      for name in nameList: 
1021          if isHierarchicalName(name): 
1022              hier_parts = name.split('.') 
1023              searchName = hier_parts[level] 
1024          else: 
1025              searchName = name 
1026          subName_parts = searchName.split('_') 
1027          if invert: 
1028              if subName != subName_parts[position]: 
1029                  matchList.append(name) 
1030          else: 
1031              if subName == subName_parts[position]: 
1032                  matchList.append(name) 
1033      return matchList

1034  
 
1035  
 


1036 -def searchModelSpec(mspec, name, component_type_order=None):


1037      """Find Quantity or sub-component objects containing named <name>
 
1038      of type given by the hierarchical name <comptype1.comptype2. ... .name>,
 
1039      where component_type_order = [<comptype1>, <comptype2>, ... ],
 
1040      and <comptypeN> may be a component type or a specific component name.""" 
1041      if isHierarchicalName(name): 
1042          # transfer root names to component_type_order
 
1043          if component_type_order is None or component_type_order == []: 
1044              # OK to continue
 
1045              parts = name.split(NAMESEP) 
1046              return searchModelSpec(mspec, parts[-1], parts[:-1]) 
1047          else: 
1048              raise ValueError("Cannot pass both a hierarchical name to search"
 
1049                               " and a non-empty component_type_order argument") 
1050      if component_type_order is None or component_type_order == []: 
1051          # we have gone as far as intended
 
1052          if name in mspec._registry: 
1053              return [name] 
1054          elif name in mspec.components: 
1055              return [name] 
1056          elif name in mspec._componentTypeMap: 
1057              return [mspec._componentNameMap[o] for o in \
 
1058                      mspec._componentTypeMap[name]] 
1059          else: 
1060              return [] 
1061      else: 
1062          ct = component_type_order[0] 
1063          try: 
1064              complist = mspec._componentTypeMap[ct] 
1065          except KeyError: 
1066              # ct not present in this generator as a type
 
1067              # see if it's an actual component
 
1068              try: 
1069                  complist = [mspec.components[ct]] 
1070              except KeyError: 
1071                  return [] 
1072          result = [] 
1073          # special case for distinguishing RHSfuncSpec vs ExpFuncSpec vars, which
 
1074          # have to be the last component type specified
 
1075          if name in ['RHSfuncSpec', 'ExpFuncSpec'] and component_type_order[1:] == []: 
1076              for comp in complist: 
1077                  compname = mspec._componentNameMap[comp] 
1078                  try: 
1079                      test = mspec._registry[compname].obj.specType == name 
1080                  except (KeyError, AttributeError): 
1081                      # invalid kind of object to have a specType (e.g. a Component)
 
1082                      test = False 
1083                  if test: 
1084                      result.append(compname) 
1085          else: 
1086              for comp in complist: 
1087                  comp_result = searchModelSpec(comp, name, component_type_order[1:]) 
1088                  result.extend([comp.name + NAMESEP + r for r in comp_result]) 
1089          return result

1090  
 
1091  # -------------------------------------------------------------------------
 
1092  ### Quantity 'for' macro utilities for ModelSpecs
 
1093  
 


1094 -def processMacro(c, mspec, forSubs=False):


1095      """Process 'for' macro occurrences in a Quantity specification.
 
1096  
 
1097     forSubs = True causes any target names to be resolved to their
 
1098     global names and returned to caller so that they can be substituted
 
1099     textually into the Quantity's specification.""" 
1100  
 
1101      ctypestrlist = []   # component type names to remove 
1102      targList = [] 
1103      toks = c.spec.parser.tokenized 
1104      num_fors = toks.count('for') 
1105      specStr = "" 
1106      forpos = 0 
1107      for for_ix in range(num_fors): 
1108          old_forpos = forpos 
1109          forpos += toks[forpos:].index('for') 
1110          if old_forpos == 0: 
1111              specStr += "".join(toks[:forpos]) 
1112          else: 
1113              specStr += "".join(toks[old_forpos+1:forpos]) 
1114          qtemp = QuantSpec('macrospec', toks[forpos+1]) 
1115  #        targList_new, ctStr, opStr = resolveMacroTargets(qtemp.parser.tokenized,
 
1116  #                                                     registry, c.name)
 
1117          parentName = ".".join(c.name.split('.')[:-1]) 
1118          sourceList = searchModelSpec(mspec, parentName) 
1119          if len(sourceList) == 1: 
1120              sourceName = sourceList[0] 
1121          elif len(sourceList) == 0: 
1122              sourceName = "" 
1123          else: 
1124              print "Found: ", sourceList 
1125              raise ValueError("source list for macro resolution should have no "
 
1126                               "more than one entry") 
1127          targList_new, ctStr, opStr = resolveMacroTargets(qtemp.parser.tokenized,
 
1128                                                           mspec, sourceName) 
1129          ctypestrlist.append(ctStr) 
1130          if forSubs: 
1131              for t in targList_new: 
1132                  if t not in targList: targList.append(t) 
1133          if targList_new == []: 
1134              specStr += "0" 
1135          else: 
1136              specStr += "(" + opStr.join(targList_new) + ")" 
1137          forpos += 1 
1138      # add rest of original spec string
 
1139      specStr += "".join(toks[forpos+1:]) 
1140      c.spec = QuantSpec(replaceSep(c.spec.subjectToken), specStr.strip(),
 
1141                         c.spec.specType) 
1142      # remove componentTypeStr from c.freeSymbols and
 
1143      # c.usedSymbols
 
1144      c.freeSymbols = remain(c.freeSymbols, ctypestrlist) 
1145      c.usedSymbols = remain(c.usedSymbols, ctypestrlist+['for']) 
1146      return (c, targList, ctypestrlist)

1147  
 
1148  
 


1149 -def resolveMacroTargets(toks, mspec, targParent):


1150      # opStr already checked to be either + or *
 
1151      opStr = toks[5] 
1152      localTarget = toks[3] 
1153      componentTypeStr = toks[1] 
1154      if componentTypeStr in ['Var', 'Par', 'Quantity']: 
1155          raise ValueError("Invalid component type for macro in"
 
1156                           " %s"%targParent) 
1157      if targParent == "": 
1158          target = componentTypeStr+'.'+localTarget 
1159      else: 
1160          target = targParent+'.'+componentTypeStr+'.'+localTarget 
1161      targList = searchModelSpec(mspec, target) 
1162      return (targList, componentTypeStr, opStr)

1163  
 
1164  # ---------------------------------------------------------------------------
 
1165  ### Private classes
 
1166  
 


1167 -class regObject(object):


1168      """Registry object container.""" 
1169  
 


1170 -    def __init__(self, obj, parent, namemap={}):


1171          self.obj = obj 
1172          self.objClass = obj.__class__ 
1173          self.objParentClass = parent.__class__ 
1174          self.objParentName = parent.name 
1175          self.objLocalName = obj.name 
1176          # maps local variable name to globalized name (if global, o/w identical)
 
1177          self.namemap = copy(namemap)

1178  
 


1179 -    def __eq__(self, other):


1180          tests = [] 
1181          try: 
1182              tests.append(self.objClass == other.objClass) 
1183              tests.append(self.objParentClass == other.objParentClass) 
1184              tests.append(self.objParentName == other.objParentName) 
1185              tests.append(self.objLocalName == other.objLocalName) 
1186              tests.append(self.obj == other.obj) 
1187              tests.append(self.namemap == other.namemap) 
1188          except AttributeError: 
1189              return False 
1190          return alltrue(tests)

1191  
 


1192 -    def __repr__(self):


1193          return "regObject %s (%s)"%(self.objLocalName, className(self.obj))

1194  
 


1195 -    def __str__(self):


1196          return self.obj.spec()

1197  
 


1198 -    def __call__(self):


1199          return (self.objLocalName, self.objClass, self.namemap, self.obj)

1200  
 


1201 -    def getGlobalName(self):


1202          if self.namemap == {}: 
1203              return self.objLocalName 
1204          else: 
1205              try: 
1206                  return self.namemap[self.objLocalName] 
1207              except KeyError: 
1208                  print "Namemap for '%s' is: "%self.__repr__(), self.namemap 
1209                  raise ValueError("Namemap for registry object didn't "
 
1210                                   "contain object's name "
 
1211                                   "'%s'"%self.objLocalName)

1212  
 
1213  
 


1214 -class typeCounter(object):


1215 -    def __init__(self):


1216          self.varcount = self.parcount = self.funcount = self.inpcount = 0

1217  
 


1218 -    def __getitem__(self, typelabel):


1219          if typelabel == 'var': 
1220              return self.varcount 
1221          elif typelabel == 'par': 
1222              return self.parcount 
1223          elif typelabel == 'input': 
1224              return self.inpcount 
1225          elif typelabel == 'auxfn': 
1226              return self.funcount 
1227          else: 
1228              raise KeyError("Invalid key")

1229  
 


1230 -    def __setitem__(self, typelabel, value):


1231          if typelabel == 'var': 
1232              self.varcount = value 
1233          elif typelabel == 'par': 
1234              self.parcount = value 
1235          elif typelabel == 'input': 
1236              self.inpcount = value 
1237          elif typelabel == 'auxfn': 
1238              self.funcount = value 
1239          else: 
1240              raise KeyError("Invalid key")

1241  
 
1242  
 
1243  
 


1244 -class nameResolverClass(object):


1245      """This class keeps a tab of how many times a local name has been
 
1246     used for a given specfication type ('var', 'par', 'input' or
 
1247     'auxfn'), and renames it with an appropriate globalized name
 
1248     (hierarchical according to declared parent object, with possible
 
1249     numbered suffix for multiple name declarations).
 
1250  
 
1251     Only one instance of this class is needed for a session.""" 
1252  
 


1253 -    def __init__(self):


1254          self.database = {}

1255  
 


1256 -    def __call__(self, obj, fspec, parentname=None):


1257          typelabel = className(obj).lower() 
1258          if parentname is None: 
1259              fullname = obj.name 
1260          else: 
1261              # create hierarchical name
 
1262              fullname = parentname + NAMESEP + obj.name 
1263          if fspec.name not in self.database: 
1264              self.database[fspec.name] = {} 
1265          if obj.name in self.database[fspec.name]: 
1266              self.database[fspec.name][fullname][typelabel] += 1 
1267              namecount = self.database[fspec.name][fullname][typelabel] 
1268          else: 
1269              self.database[fullname] = typeCounter() 
1270              namecount = 0 
1271          if namecount == 0: 
1272              globalname =  fullname 
1273          else: 
1274              # add number to end of name if multiple instances of the
 
1275              # same hierarchical name declared
 
1276              globalname =  fullname + NAMESEP + str(namecount) 
1277          return globalname

1278  
 


1279 -    def __repr__(self):


1280          return "ModelSpec internal helper class: nameResolver object"

1281  
 
1282      __str__ = __repr__ 
1283  
 


1284 -    def clear(self, fspec):


1285          if fspec.name in self.database: 
1286              del self.database[fspec.name]

1287  
 


1288 -    def clearall(self):


1289          self.database = {}

1290  
 
1291  
 
1292  # use single instance of nameResolver per session
 
1293  global nameResolver 
1294  nameResolver = nameResolverClass() 
1295
```

  


| Home | Trees | Indices | Help | PyDSTool |
| --- | --- | --- | --- | --- |

|  |  |
| --- | --- |
| Generated by Epydoc 3.0.1 on Fri May 4 15:24:20 2012 | http://epydoc.sourceforge.net |
